# Using direct method to estimate calcium digestibility of barley and soybean meal in quail chicks

**DOI:** 10.1016/j.psj.2025.104775

**Published:** 2025-01-04

**Authors:** Hadis Khajali, Mahmoud Ghazaghi, Mohammad Rokouei, Mehran Mehri

**Affiliations:** Department of Animal Sciences, Faculty of Agriculture, University of Zabol, Sistan 98661-5538, Iran

**Keywords:** Barley, Calcium, Endogenous loss, Direct method, Digestibility

## Abstract

The availability of calcium (Ca) in poultry diets is influenced by various factors, such as the feed ingredients used. This study assessed the apparent ileal digestibility (AID) and standardized ileal digestibility (SID) of Ca in barley and soybean meal (SBM) in young quail chicks using a direct method. Three diets were formulated, including a Ca-free basal diet to evaluate ileal endogenous calcium losses (IECaL), and two diets with barley or SBM as the sole Ca sources. Titanium dioxide was used as an indigestible marker for IECaL measurement. On day 30, 300 male quail chicks were distributed into the three dietary treatments, and ileal digesta samples were collected for Ca content analysis on day 34. Endogenous ileal Ca loss was determined to be 336 ± 52.7 mg/kg of dry matter intake (DMI), providing a reference for calculating SID values. The AID and SID of Ca in barley were found to be 27.9 % and 39.9 %, respectively, and for SBM, these values were 33.1 % and 42.9 %, respectively. These findings offer valuable insights into how to optimize poultry diets to ensure they receive adequate calcium, which is crucial for their overall health and productivity.

## Introduction

Calcium (Ca) is a critical nutrient in the diet of growing quails, playing a vital role in skeletal development and overall growth performance. The importance of Ca nutrition in quails is underscored by its involvement in bone mineralization and eggshell formation, which are essential for the health and productivity of these birds (Silva et al., 2009). Adequate Ca intake is necessary to support the rapid growth rates observed in quails, as Ca is a major component of the avian skeletal system (Blom and Lilja, 2004).

The determination of Ca digestibility coefficients, particularly the standardized ileal digestibility of Ca (SIDC), is crucial in avian nutrition. SID provides a more accurate measure of the bioavailability of Ca from different dietary sources, allowing for precise formulation of diets to meet the specific needs of birds (David, et al., 2019). This is important in poultry nutrition, where the interaction between Ca and phosphorus (P) is well-documented. An imbalance in the dietary ratio of these minerals can lead to impaired phosphorus digestibility, which is a concern given phosphorus's role in energy metabolism and bone health (David, et al., 2023).

In quails, the necessity of determining SIDC is further emphasized by the potential negative effects of overfeeding Ca. Excessive Ca intake can lead to reduced phosphorus availability, as Ca can form insoluble complexes with phosphorus in the gut, hindering its absorption (Lee and Kong, 2024). This interaction necessitates careful balancing of Ca and phosphorus in quail diets to prevent nutritional imbalances that could impair growth and development.

The application of SIDC in diet formulation for quails is essential to avoid the detrimental effects of Ca overfeeding. By accurately assessing the digestibility of Ca, nutritionists can formulate diets that provide adequate Ca without exceeding the birds' requirements, thus preventing the negative impact on phosphorus digestibility. This approach not only supports optimal growth and bone health in quails but also enhances feed efficiency and reduces the risk of environmental pollution from excess phosphorus excretion. The importance of Ca nutrition in growing quails cannot be overstated. The determination of Ca digestibility coefficients, particularly SIDC, is a critical tool in ensuring that quail diets are both nutritionally adequate and environmentally sustainable. By preventing the overfeeding of Ca and its associated negative effects on phosphorus digestibility, SIDC plays a pivotal role in optimizing the health and productivity of quails.

This study aimed to evaluate the apparent ileal digestibility (AID) and SID of Ca in barley and soybean meal (SBM) for growing Japanese quail from 30 to 34 days post-hatch, using the direct method and measuring ileal endogenous Ca losses (IECaL) by feeding a Ca-free diet.

## Materials and methods

### Ethics statement

This study's protocol received approval from the University of Zabol's Research Animal Ethics (AEUOZ-2012-BR) Committee and complied with the guidelines established by the Iranian Council for Animal Care.

### Chemical analysis

Prior to the experiment, all feed ingredients used in the experimental diets including corn, barley, and SBM, were analyzed for dry matter (DM; method 930.15, AOAC, 2006), ash content (method 942.05, AOAC, 2006), crude fiber (method 978.10, AOAC, 2006), ether extract (method 2003.05, AOAC, 2006), Ca (method 934.01,AOAC (2006), and crude protein (CP; method 990.03, AOAC, 2006).

### Birds Management and Experimental Diets

In this digestibility study, 300 quail chicks were used from 30 to 34 days post-hatch to assess the AID and SID of Ca in barley and SBM. Until 29 days of age, chicks were fed a standard diet formulated to meet the nutrient needs of growing Japanese quail, following NRC (1994) guidelines. Three dietary treatments were prepared: a Ca-free diet, barley, and SBM diets, with each treatment replicated five times and 20 birds per floor pen (length = 100 cm, width = 92.9 cm, and height = 60 cm). A corn-based Ca-free diet was used to determine the IECaL, and the experimental diets were formulated with either barley or SBM as the sole Ca sources (Table 1). Titanium dioxide (5 g/kg) served as the indigestible marker in each diet. At the end of the study, birds were euthanized via CO_2_ asphyxiation to collect ileal digesta by flushing the distal two-thirds of the ileum with distilled water. Digesta from each pen were pooled and stored at −20 °C for later analysis. Bird performance, including feed intake, body weight, and feed conversion ratio, was measured during the study.

**Table 1:** Composition of the experimental diets.

Ingredient	Amount (%)		
	Ca-free diet	Barley	Soybean meal
Test ingredient	-	97.07	45.00
Corn, grain	97.00	-	51.08
Soybean oil	1.00	1.00	2.55
NaH_2_PO_4_	-	-	0.05
KHCO_3_	0.62	0.54	-
TiO_2_	0.50	0.50	0.50
NaCl	0.32	0.14	0.31
Mineral Premix^1^	0.25	0.25	0.25
Vitamin Premix^2^	0.25	0.25	0.25
NaHCO_3_	0.06	0.25	-
Nutrient composition			
AME (Kcal/kg)	3337	2651	3000
CP (%)	7.68	11.0	25.9
Lysine (%)	0.23	0.38	1.43
Methionine (%)	0.16	0.17	0.37
Methionine + Cysteine (%)	0.33	0.35	0.77
Threonine (%)	0.28	0.41	0.99
Tryptophan (%)	0.06	0.14	0.31
Ca (%)	0.02	0.06	0.14
P available (%)	0.10	0.16	0.20
Na (%)	0.16	0.16	0.16
K (%)	0.91	1.00	1.05
Cl (%)	0.23	0.23	0.23
DEB (mEq/kg)^3^	79.2	124	270

1 Mineral premix provided per kilogram of diet: Mn (from MnSO4·H2O), 65 mg; Zn (from ZnO), 55 mg; Fe (from FeSO4·7H2O), 50 mg; Cu (from CuSO4·5H2O), 8 mg; I [from Ca (IO3)2·H2O], 1.8 mg; Se, 0.30 mg; Co (from Co2O3), 0.20 mg; Mo, 0.16 mg.

2 Vitamin premix provided per kilogram of diet: vitamin A (from vitamin A acetate), 11,500 IU; cholecalciferol, 2,100 IU; vitamin E (from dl-α-tocopheryl acetate), 22 IU; vitamin B12, 0.60 mg; riboflavin, 4.4 mg; nicotinamide, 40 mg; calcium pantothenate, 35 mg; menadione (from menadione dimethylpyrimidinol), 1.50 mg; folic acid, 0.80 mg; thiamine, 3 mg; pyridoxine, 10 mg; biotin, 1 mg; choline chloride, 560 mg; ethoxyquin, 125 mg.

3 Dietary Electrolyte Balance: represents dietary Na +*K* − Cl in mEq/kg of diet.

### Calculations

Following the method described by Anwar, et al. (2016), the AID coefficient of Ca was determined using the indigestible marker ratio between diets and digesta:

$$AIDC=1-[\left(Ti_{D}/Ti_{I}\right)\times \left(Ca_{I}/Ca_{D}\right)]$$

where AIDC represents the apparent ileal digestibility coefficient of Ca, while Ti_D_, Ti_I_, Ca_I_, and Ca_D_ correspond to the titanium concentration in the diet, titanium concentration in the ileal digesta, Ca concentration in the ileal digesta, and Ca concentration in the diet, respectively. The analyzed concentrations were all reported in grams per kilogram of dry matter (DM).

Ileal endogenous Ca losses (IECaL; g/kg DM intake) were estimated as follows:

$$IECaL=Ca_{O}\times \left(Ti_{I}/Ti_{D}\right)$$

where IECaL represents ileal endogenous Ca losses, while Ti_D_, Ti_I_, and Ca_O_ denote the titanium concentration in the diet, the ileal digesta, and the Ca concentration in the ileal digesta, respectively.

Standardized ileal digestibility coefficients of Ca (SIDC) of the test diets were measured as follows:

$$SIDC=AIDC+\left[IECaL\left(g/kgofDMI\right)/Ca_{D}\left(g/kgofDM\right)\right]$$

where AIDC and SIDC stand for apparent and standardized ileal digestibility coefficients of Ca, respectively. IECaL indicates ileal endogenous Ca losses (g/kg of DMI), and Ca_D_ refers to the Ca concentration in the diet (g/kg of DM).

### Statistical analysis

A one-way ANOVA in SAS (2002), using the General Linear Model (GLM) procedure, was used to analyze the data. Pen means served as the experimental unit. Using a significance level of *P* < 0.05, the LSD test applied to determine statistically significant differences between the means.

## Result and discussion

**Fig. 1:**
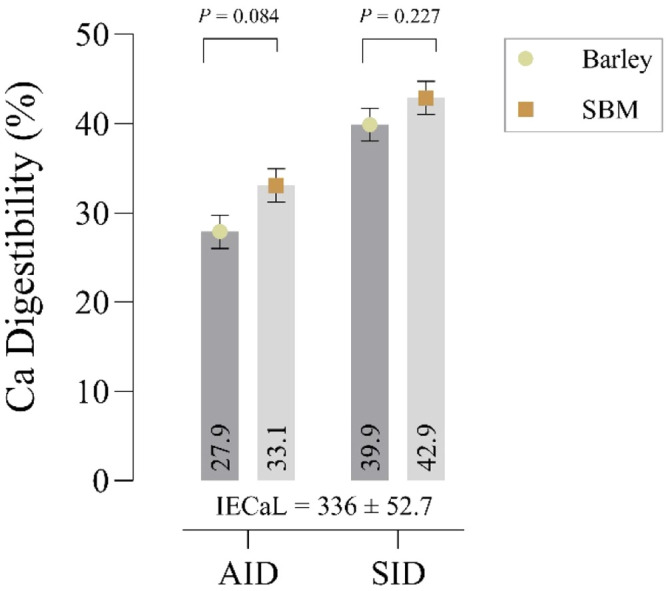
Apparent ileal digestibility (AID ± SEM), standardized ileal digestibility (SID ± SEM), ileal endogenous losses (IECaL; mg/kg DMI) of calcium in growing quail chicks.

The AIDC in barley was 27.9 %, which was tended to be lower (*P* = 0.084) than that in SBM (33.1 %; Fig. 1). In contrast, the SIDC in barley (39.9 %) and SBM (42.9 %) did not show significant difference (*P* = 0.227), and IECaL was estimated at 336 ± 52.7 mg/kg of DMI.

The determination of IECaL is a critical factor in calculating SID values (Adeola, et al., 2016), which are essential for accurate nutritional assessments in poultry and refers to non-dietary components present in the digesta at the terminal ileum of poultry, which comprise digestive secretions, mucus, and shed gut epithelial cells. In this study, the IECaL was determined to be 336 ± 52.7 mg/kg of dry matter intake (DMI), which was higher than that obtained in another study on growing quails in our lab (179 ± 68.6 mg/kg of DMI; not published). There are some factors that may cause this difference in IECaL between two studies. Although limited information is available on ileal endogenous calcium losses (IECaL), studies have shown that various factors—such as the type of experimental animal, age, sex, feed intake, and the ingredient composition of the basal diet—can influence ileal endogenous amino acid losses. It is plausible that these factors may similarly affect IECaL through comparable mechanisms (Adeola, et al., 2016).

The AID and SID of Ca in barley were found to be 27.9 % and 39.9 %, respectively, while for SBM, these values were 33.1 % and 42.9 %. These results highlight the differences in AIDC coefficients between feed ingredients (*P* = 0.084) can be attributed to their inherent nutritional composition and the presence of antinutritional factors (Anwar, et al., 2016), while the correction for IECaL revealed that the use of AIDC lead to underestimation of the true digestibility (David, et al., 2019) and SIDC coefficients for both test ingredients (i.e., barley and SBM) were not significantly different. The determination of SIDC coefficients for feed ingredients is crucial in this context, as it allows for precise formulation of diets that meet the specific needs of birds without exceeding their calcium requirements, since the excessive dietary Ca can impair P absorption by forming insoluble complexes in the gut, thereby reducing its bioavailability (David, et al., 2023).

The results of studies on broiler chickens underscore the necessity of using SIDC values in a diet formulation for quails to avoid the detrimental effects of Ca overfeeding. By accurately assessing the digestibility of Ca, nutritionists can formulate diets that provide adequate Ca without exceeding the birds' requirements, thus preventing the negative impact on P digestibility.

In conclusion, the determination of IECaL and the estimation of SIDC coefficients are essential components of accurate nutritional assessments of Ca resources in poultry. The results of this study provide valuable insights into the digestibility of Ca in barley and SBM, highlighting the importance of accounting for IECaL in digestibility calculations. Although the AIDC in barley (27.9 %) and SBM (33.1 %) showed a tendency, the SIDC values proved that growing quail chicks could digest these ingredients for Ca equally. Due to the crucial interaction between Ca and P in poultry species, it is essential to shift from using total calcium to digestible calcium in quail feed formulations. This transition aims to prevent nutritional imbalances, optimizing the health and productivity of quails.

## Declaration of competing interest

The authors whose names are listed immediately below certify that they have NO affiliations with or involvement in any organization or entity with any financial interest (such as honoraria; educational grants; participation in speakers’ bureaus; membership, employment, consultancies, stock ownership, or other equity interest; and expert testimony or patent-licensing arrangements), or non-financial interest (such as personal or professional relationships, affiliations, knowledge or beliefs) in the subject matter or materials discussed in this manuscript.
